# The changing landscape of substance use disorders over 30 years: insights on US state disparities and policy from the global burden of disease study

**DOI:** 10.1186/s12963-026-00476-3

**Published:** 2026-04-20

**Authors:** Shahrzad Bazargan-Hejazi, Wendy Shang, Najmeh Mohammadi, Kaveh Dehghan, Sanam Ahadi, Misagh Naderi, Anaheed Shirazi

**Affiliations:** 1 Alice L. Walton School of Medicine, 1001, NE J Street, Bentonville, 72712 AR USA; 2 Department of Psychiatry and Biobehavioral Sciences, University of California, Los Angeles, Los Angeles, CA USA; 3 Department of Psychiatry, College of Medicine, Charles R. Drew University of Medicine and Science, Los Angeles, CA USA; 4 Department of Health and Life Sciences, Charles R. Drew University of Medicine and Science, Los Angeles, CA USA; 5 Department of Mathematics and Computer Science, Amirkabir University of Technology, Tehran, Iran; 6 College of Medicine, Charles R. Drew University of Medicine and Science, Los Angeles, CA USA; 7 Department of Psychiatry, University of California, San Diego, San Diego, CA USA

**Keywords:** Substance use disorders, Disability-adjusted life years, Global burden of disease study, Opioid use disorder, Alcohol use disorder, Health policy, United States

## Abstract

**Background:**

Substance use disorders (SUDs) remain a major source of preventable morbidity and mortality in the United States. This study described trends in the burden of SUDs from 1990 to 2019 by substance, sex, and age, and examined whether state-level policy environments and behavioral health budgets differed across states with the highest and lowest SUD-related disability-adjusted life years (DALYs).

**Methods:**

We conducted a descriptive epidemiologic study using Global Burden of Disease Study 2019 estimates for alcohol, opioid, cocaine, amphetamine, cannabis, and other drug use disorders in the United States. DALYs were the primary outcome and were examined by sex, age, substance, and year. To contextualize state-level disparities, we descriptively summarized behavioral health budget allocations and selected policy domains in states with the three highest and three lowest DALY rates for drug use disorders and alcohol use disorders.

**Results:**

From 1990 to 2019, prevalent SUD cases in the United States increased from 12.6 million to 19.5 million, and the age-standardized DALY rate for SUDs rose from 725.5 to 2,274.4 per 100,000 population. Opioid use disorders showed the largest increases in both prevalence (618.5%) and DALY rates (643.7%), becoming the leading contributor to SUD-related burden. Cocaine and amphetamine use disorders showed smaller increases in prevalence but larger increases in DALY rates, whereas alcohol use disorder changed modestly and cannabis-related DALY rates remained unchanged. In 2019, the age-standardized DALY rate for SUDs was higher among males than females (2,486.8 vs. 1,722.6 per 100,000 population). Opioid-related DALYs peaked in early adulthood, whereas alcohol-related DALYs peaked in midlife. Substantial geographic variation was observed: drug-related DALY rates were highest in West Virginia, Kentucky, and Ohio and lowest in Nebraska, South Dakota, and North Dakota, while alcohol-related DALY rates were highest in New Mexico, Alaska, and the District of Columbia and lowest in New Jersey, Maryland, and Texas. States with lower burden generally reflected more prevention-oriented and coordinated policy environments, although the presence of policy alone did not consistently correspond to lower burden.

**Conclusions:**

The burden of SUDs in the United States increased substantially over three decades, driven primarily by opioids and with marked variation across sex, age, and geography. Descriptive comparisons suggest that policy context and behavioral health investment may help shape state-level differences, but implementation, treatment access, and broader structural conditions also matter. Coordinated, equitable, and adequately resourced prevention, treatment, and harm-reduction strategies are needed to reduce persistent disparities in SUD-related outcomes.

**Supplementary Information:**

The online version contains supplementary material available at 10.1186/s12963-026-00476-3.

## Background

The World Health Organization categorizes substance use disorders (SUDs) as conditions involving the harmful use of psychoactive substances, covering both SUDs and dependence [1, 2]. We use the term SUDs across this study, to reflect a continuum of severity, from mild to severe. This is consistent with the DSM-5 replacing categories of substance abuse and substance dependence with a single diagnosis, substance use disorder. This shift aligns with contemporary clinical practice and public health efforts to reduce stigma and improve engagement with care [3, 4]. 

The burdens associated with SUDs extend across physical, mental, social, and economic dimensions, reflecting the intricate interplay of individual and societal factors. Individuals with SUDs face elevated risks of infectious diseases, such as blood-borne viruses and hepatitis C [5, 6]. along with non-communicable diseases like chronic obstructive pulmonary disease, heart disease, and diabetes [7, 8]. Additionally, both fatal and non-fatal suicidal behaviors, including suicide deaths, suicide attempts, and suicidal ideation, along with intimate partner violence, homelessness, incarceration, violent encounters, and family instability, further exacerbate the mental and societal toll of SUDs [7, 9–13]. Furthermore, SUDs impose substantial economic challenges, encompassing the financial impact of crime, productivity losses, and escalating healthcare costs [14]. The estimated annual economic impact surpasses $650 billion [15, 16], with potential escalation to nearly $1 trillion [17]. These problems contribute to the global burden of disease, measured by Disability-Adjusted Life Years (DALYs), which involves the burden due to disability through Years Lived with Disability (YLDs) and Years of Life Lost (YLLs) to premature mortality [18–20]. 

According to the Degenhardt et al. [5] study, substance use in the United States is among the highest globally. The United States surpasses other regions, including East Asia, South Asia, and Eastern Europe, in terms of the prevalence of cannabis, cocaine, and opioid dependence. Within the states, the prevalence of SUDs has shown an upward trend over the years [20–22]. Findings from the National Survey of Drug Use and Health (NSDUH) reveal a notable increase in the use of illicit drugs across all age groups from 2018 to 2019, except for young adults. Although NSDUH has documented age-related trends in SUDs, it primarily focuses on self-reported prevalence and does not quantify the broader health burden of SUDs [23]. Our use of DALYs incorporates both fatal and nonfatal outcomes, providing a more comprehensive measure of the population health burden attributable to SUDs.

Concurrently, there has been a general decline in alcohol consumption among various age cohorts, although adolescents are an exception to this pattern [24, 25]. Also, existing reports point to shifts in substance use patterns over the past decade. While alcohol misuse was more prevalent from 2007 to 2015, recent reports point to a transition toward the use of illicit drugs in subsequent years [26, 27]. This dynamic landscape of substance use and the complex interaction between physical, social, and economic dimensions underscores the need for ongoing research and targeted interventions to address evolving trends, ensuring that public health strategies remain adaptive and effective in response to the changing nature of substance misuse.

Despite national-level goals set by Healthy People 2030, the burden of SUDs remains unevenly distributed across the United States [28, 29]. Substance use disorder outcomes are not solely driven by individual behaviors but are shaped by broader social and policy environments [30–32]. State-level drug policies may influence the prevalence and burden of SUDs by shaping access to treatment, regulating the availability of substances, and altering social norms. This study aims [1] to describe trends in the burden of SUDs, including opioids, alcohol, cannabis, cocaine, and amphetamines, in the United States from 1990 to 2019 by sex, age, and year using Global Burden of Disease Study 2019 (GBD 2019) [2], to explore how differences in state-level public health policies and behavioral health budgets align with the three states with the highest and lowest SUD-related DALYs. These policies and funding frameworks are analyzed against national benchmarks, such as those set by Healthy People 2030 [28]. By comparing states with the highest and lowest burdens of drug and alcohol use disorders, this study provides insights into evolving SUD trends, demographic disparities, and the potential influence of policy environments and resource allocation on health outcomes.

## Materials and methods

### Data source, estimates, and measures

This was a descriptive epidemiology study using GBD 2019 estimates. GBD 2019 provides annual estimates from 1990 to 2019 of mortality and health loss (YLLs, YLDs, and DALYs) for 369 diseases and injuries and 286 causes of death, and attributable burden for 87 risk factors, across 204 countries and territories. Within this framework, the term “cause” refers to standardized disease or injury categories used to measure population health burden and does not imply direct causality. For example, SUDs and cardiovascular diseases are classified as distinct causes contributing to DALYs without asserting etiological linkage [33]. 

DALY estimates are generated through a rigorous, standardized modeling process that integrates data from epidemiological studies, population-based surveys, administrative and claims datasets, and vital registration systems. Fatal outcomes, expressed as YLL, are estimated using the Cause of Death Ensemble model (CODEm), while non-fatal outcomes, expressed as YLDs, are estimated using Bayesian meta-regression through the DisMod-MR 2.1 tool [33–35]. For all estimates, 95% uncertainty intervals (UIs) are calculated as the 2.5th and 97.5th percentile values across 1,000 posterior draws [36]. 

Although the GBD study incorporates data from the National Survey on Drug Use and Health (NSDUH) as one of its inputs, it differs from NSDUH by synthesizing multiple data sources, applying standardized disability weights, and modeling disease burden across time, age, sex, and geography. As such, the GBD study complements NSDUH by providing a comprehensive measure of population health loss attributable to SUDs, rather than prevalence alone. A detailed summary of primary data resources informing the GBD SUD estimates, along with their respective strengths and limitations, is provided in Supplemental (S) Table 1.

Using GBD 2019 estimates, we assessed SUD burden by sex and age across alcohol, opioid, cocaine, amphetamine, cannabis, and other drug use disorders. Case definitions draw on DSM-IV/ICD-10 crosswalks; therefore, we use DSM-IV- consistent, non-stigmatizing SUD terminology throughout. We also descriptively examined state-level prevention efforts, public health policies, and behavioral health budget allocations.

State-level policy data are drawn from reports published between 2019 and 2020 by Trust for America’s Health (TFAH), the Alcohol Policy Information System, and relevant state legislative documents [37–39]. Budget data reflect publicly available state allocations for behavioral and mental health services during fiscal years 2019 or 2020, depending on source availability, including reports from the National Association of State Mental Health Program Directors [40]. 

Policy domains examined included: Good Samaritan laws, naloxone access, prescription drug monitoring programs (PDMPs), Social Host Liability laws, Sunday Sale Bans (i.e., time of alcohol sales), and Dram Shop Liability statutes (i.e., serving responsibility to prevent harm from overservice). These were selected based on their documented roles in overdose prevention, harm reduction, and regulatory oversight [28]. The use of these policy domains also allowed for consistent comparison of policy environments across states with the highest and lowest SUD-related DALY burdens.

### Data reporting plan/statistical analysis

Using the Python Programming Language, we employed the Jupyter Notebook interface to report SUDs’ burdens (DALYs) by sex, age groups, and years. Outputs included frequency/counts, percentage, rates, graphs, maps, and tables with uncertainty intervals. 95% UIs were calculated as the 2.5th and 97.5th percentile values across 1,000 draws [36]. 

## Results

### National prevalence and age-standardized DALY rates for substance use disorders, 1990–2019

As illustrated in Table 1, prevalent SUD cases in the United States increased from 12.6 million (95% UI 11.1–14.4) in 1990 to 19.5 million (17.9–21.2) in 2019, a 54.4% increase over the study period. During the same period, the age-standardized DALY rate attributable to SUDs increased by 213.5%, from 725.5 per 100,000 population (95% UI 563.0–875.1.0.1) to 2,274.4 per 100,000 (1,947.1-2.1,640.6).

**Table 1:** Prevalence and age-standardized DALY rates for substance use disorders in the United States, 1990–2019

Substance	Prevalence, 1990 million (95% UI)	Prevalence, 2019 million (95% UI)	Prevalence % change	Age-standardized DALY rate, 1990 (95% UI)	Age-standardized DALY rate, 2019 (95% UI)	DALY rate % change
Alcohol use disorders	7.77 (6.41–9.26)	8.20 (7.22–9.26)	5.6	376.4 (291.9–486.3.9.3)	393.2 (320.5–485.7.5.7)	4.5
Amphetamine use disorders	0.62 (0.40–1.05)	0.87 (0.65–1.12)	40.3	33.0 (20.1–49.9)	98.7 (74.9–126.5.9.5)	199.1
Cannabis use disorders	2.47 (1.83–3.19)	2.88 (2.15–3.57)	16.8	28.1 (16.3–43.6)	28.1 (16.6–44.4)	0.0
Cocaine use disorders	1.11 (0.84–1.45)	1.67 (1.37–2.02)	50.5	68.4 (45.0–93.8.0.8)	165.5 (126.5–204.0)	142.0
Drug use disorders	5.11 (4.31–5.97)	11.73 (10.67–13.00.67.00)	129.5	349.1 (269.9–416.0)	1,881.3 (1,600.8-2.8,195.5)	438.9
Opioid use disorders	0.89 (0.75–1.03)	6.36 (5.61–7.16)	618.5	204.8 (157.5–248.4.5.4)	1,523.1 (1,260.2-1.2,796.0)	643.7
Other drug use disorders	0.99 (0.75–1.24)	1.92 (1.58–2.32)	94.6	14.8 (11.3–19.2)	65.9 (51.1–81.9)	344.9
Substance use disorders (total)	12.62 (11.07–14.36)	19.49 (17.88–21.16)	54.4	725.5 (563.0–875.1.0.1)	2,274.4 (1,947.1-2.1,640.6)	213.5

Values are presented as millions of prevalent cases and age-standardized DALY rates per 100,000 population, with 95% uncertainty intervals

DALY, disability-adjusted life year; UI, uncertainty intervals

When examining substance-specific patterns, the largest increases were observed for opioid use disorders, with prevalence increasing by 618.5% and the age-standardized DALY rate by 643.7%. Other drug use disorders also showed substantial increases, with prevalence rising by 129.5% and the age-standardized DALY rate by 438.9%. Cocaine and amphetamine use disorders, both categorized as stimulant use disorders, exhibited more modest increases in prevalence (+ 50.5% and + 40.3%, respectively), but markedly larger increases in age-standardized DALY rates (+ 142.0% and + 199.1%, respectively). In contrast, alcohol use disorder showed comparatively modest changes, with prevalence increasing by 5.6% and the age-standardized DALY rate by 4.5%. Cannabis use disorder showed a 16.8% increase in prevalence, whereas the age-standardized DALY rate remained unchanged (Table 1).

**Fig. 1:**
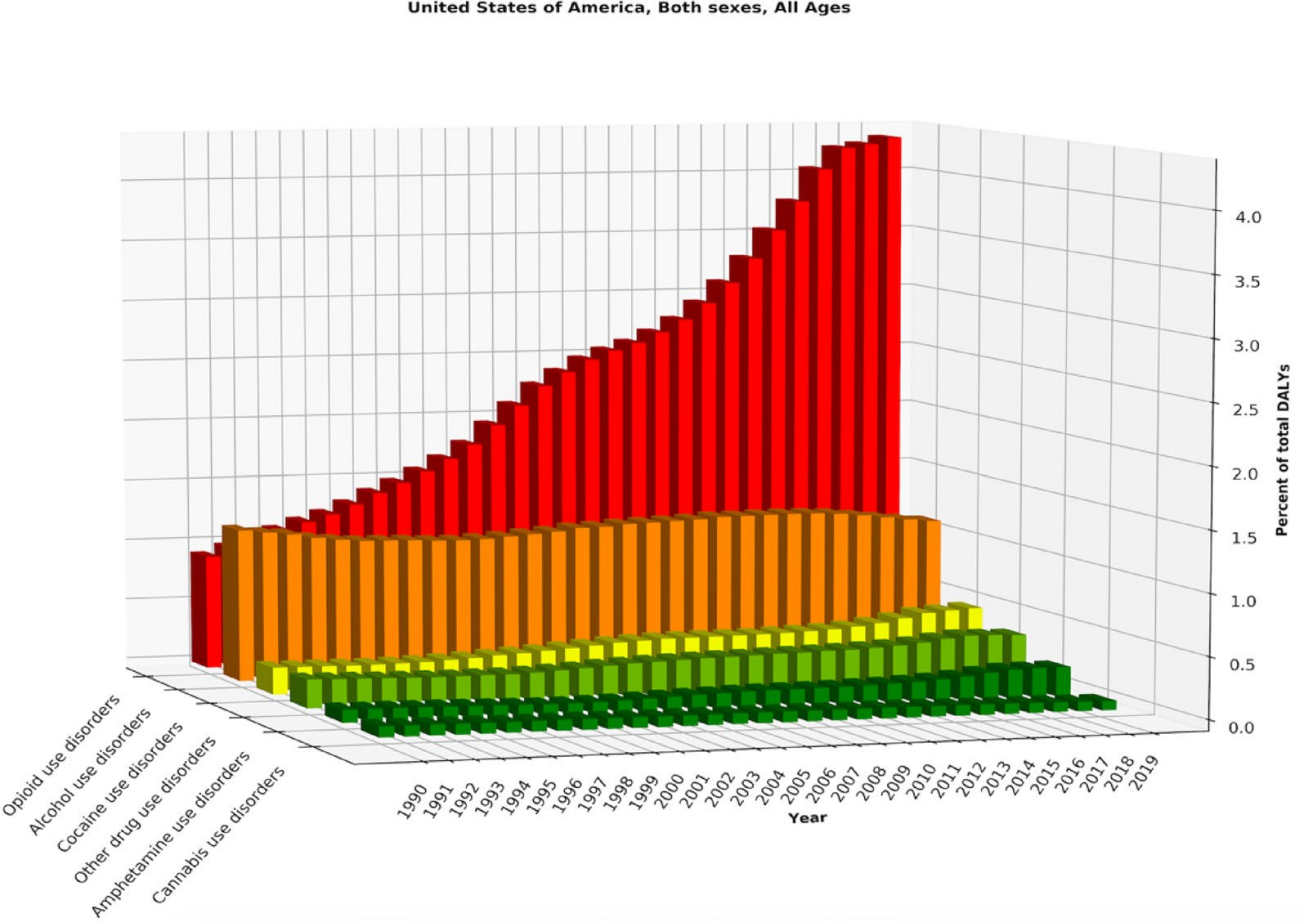
Trends in the proportion of total DALYs attributable to specific SUDs, both sexes, United States, 1990–2019

Across the study period, the proportion of total DALYs attributable to specific substance use disorders varied substantially by substance (Fig. 1). Opioid use disorders showed the most marked and sustained increase, with the sharpest growth occurring in the later years, ultimately becoming the leading contributor among SUD categories. Alcohol use disorders, by contrast, remained a consistently high contributor but showed relatively modest fluctuation over time. Cocaine and amphetamine use disorders contributed smaller proportions of total DALYs and demonstrated gradual upward trends, particularly in the latter part of the series. Other drug use disorders showed a moderate increase, whereas cannabis use disorders remained the smallest contributor throughout, with minimal change relative to the other substances (Fig. 1).

### Substance use disorder-related DALYs by sex and rate of change

Table 2 presents prevalence and age-standardized DALY rates for SUDs by sex in 1990 and 2019. Between 1990 and 2019, prevalent cases of SUDs increased among females from 4.48 million (95% UI 3.94–5.07) to 8.12 million (7.46–8.86) and among males from 8.14 million (95% UI 7.12–9.26) to 11.37 million (10.4–12.3). Over the same period, the age-standardized DALY rate increased among females from 475.6 (95% UI 358.6–586.0) to 1,722.6 (1,423.3-2.3,030.8) and among males from 1,126.2 (881.4-1.4,391.6) to 2,486.8 (2,414.6-3.6,456.3).

**Table 2:** Prevalence and age-standardized DALY rates for substance use disorders by sex, 1990 and 2019

Substance	Prevalence, 1990 million (95% UI)	Prevalence, 2019 million (95% UI)	Prevalence % change	Age-standardized DALY rate, 1990 (95% UI)	Age-standardized DALY rate, 2019 (95% UI)	DALY rate % change
**Female**						
Substance use disorders	4.48 (3.94–5.07)	8.12 (7.46–8.86)	81.3	475.6 (358.6–586.0)	1722.6 (1423.3-2030.8)	262.2
Alcohol use disorders	2.59 (2.17–3.06)	3.04 (2.66–3.46)	17.3	222.4 (164.4-296.8)	259.1 (205.5–328.0)	16.5
Opioid use disorders	0.40 (0.34–0.47)	3.21 (2.83–3.61)	699.9	149.9 (110.2-185.5)	1231.6 (983.1-1474.7)	721.5
Cocaine use disorders	0.39 (0.29–0.52)	0.56 (0.46–0.71)	45.0	45.0 (28.5–63.1)	95.9 (72.3-125.6)	112.9
Amphetamine use disorders	0.28 (0.19–0.41)	0.40 (0.31–0.51)	42.9	28.7 (16.8–43.8)	67.0 (47.9–90.4)	133.8
Cannabis use disorders	0.85 (0.62–1.11)	1.03 (0.76–1.35)	21.9	19.4 (11.1–31.1)	20.4 (11.5–32.3)	4.9
Other drug use disorders	0.05 (0.04–0.07)	0.11 (0.09–0.13)	103.3	10.1 (7.2–13.1)	48.7 (35.8–66.9)	379.7
**Male**						
Substance use disorders	8.14 (7.12–9.26)	11.37 (10.49–12.35)	39.6	1126.2 (881.4-1391.6)	2486.8 (2414.6-3456.3)	120.8
Alcohol use disorders	5.18 (4.29–6.13)	5.16 (4.55–5.81)	-0.3	538.1 (423.0-685.8)	529.2 (440.7-647.1)	-1.7
Opioid use disorders	0.49 (0.41–0.56)	3.16 (2.77–3.57)	551.3	259.6 (200.0-315.7)	1808.2 (1438.0-2166.7)	596.5
Cocaine use disorders	0.72 (0.54–0.95)	1.10 (0.91–1.32)	53.5	91.7 (59.4-125.6)	234.6 (168.7-297.2)	155.7
Amphetamine use disorders	0.34 (0.21–0.50)	0.46 (0.35–0.60)	38.1	37.3 (23.0-56.2)	130.2 (92.7-177.1)	249.3
Cannabis use disorders	1.62 (1.19–2.08)	1.85 (1.38–2.39)	14.2	36.6 (21.5–56.3)	35.6 (21.3–55.8)	-2.7
Other drug use disorders	0.04 (0.03–0.06)	0.08 (0.07–0.10)	84.2	19.5 (13.3–27.2)	82.8 (59.4-113.1)	324.5

Values are presented as millions of prevalent cases and age-standardized DALY rates per 100,000 population, with 95% uncertainty intervals

Table 2 also shows that across both sexes, opioid use disorders accounted for the largest increases in DALY rates, reaching 1,231.6 per 100,000 population (95% UI 983.1-1.1,474.7) among females and 1,808.2 per 100,000 (1,438.0–2,166.7) among males in 2019. Stimulant use disorders, including amphetamine and cocaine use disorders, also increased markedly over time, whereas DALY rates for alcohol use disorder changed modestly and those for cannabis use disorder remained relatively stable. In 2019, age-standardized DALY rates for alcohol use disorder were higher among males than females, at 529.2 per 100,000 (95% UI 440.7–647.1.7.1) versus 259.1 per 100,000 (95% UI 205.5–328.0).

### Relative changes in age-standardized rates of substance use disorder outcomes, 1990–2019

**Fig. 2:**
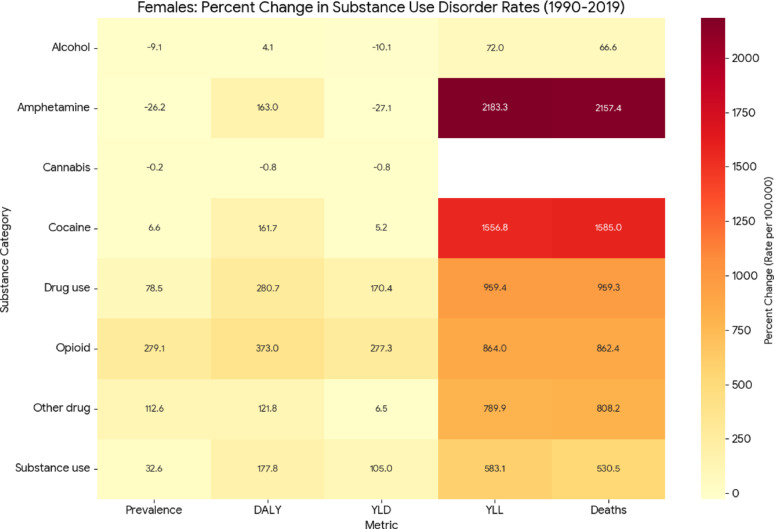
Percent change in age-standardized rates of prevalence, DALYs, YLDs, YLLs, and deaths for SUDs among females, United States, 1990–2019

**Fig. 3:**
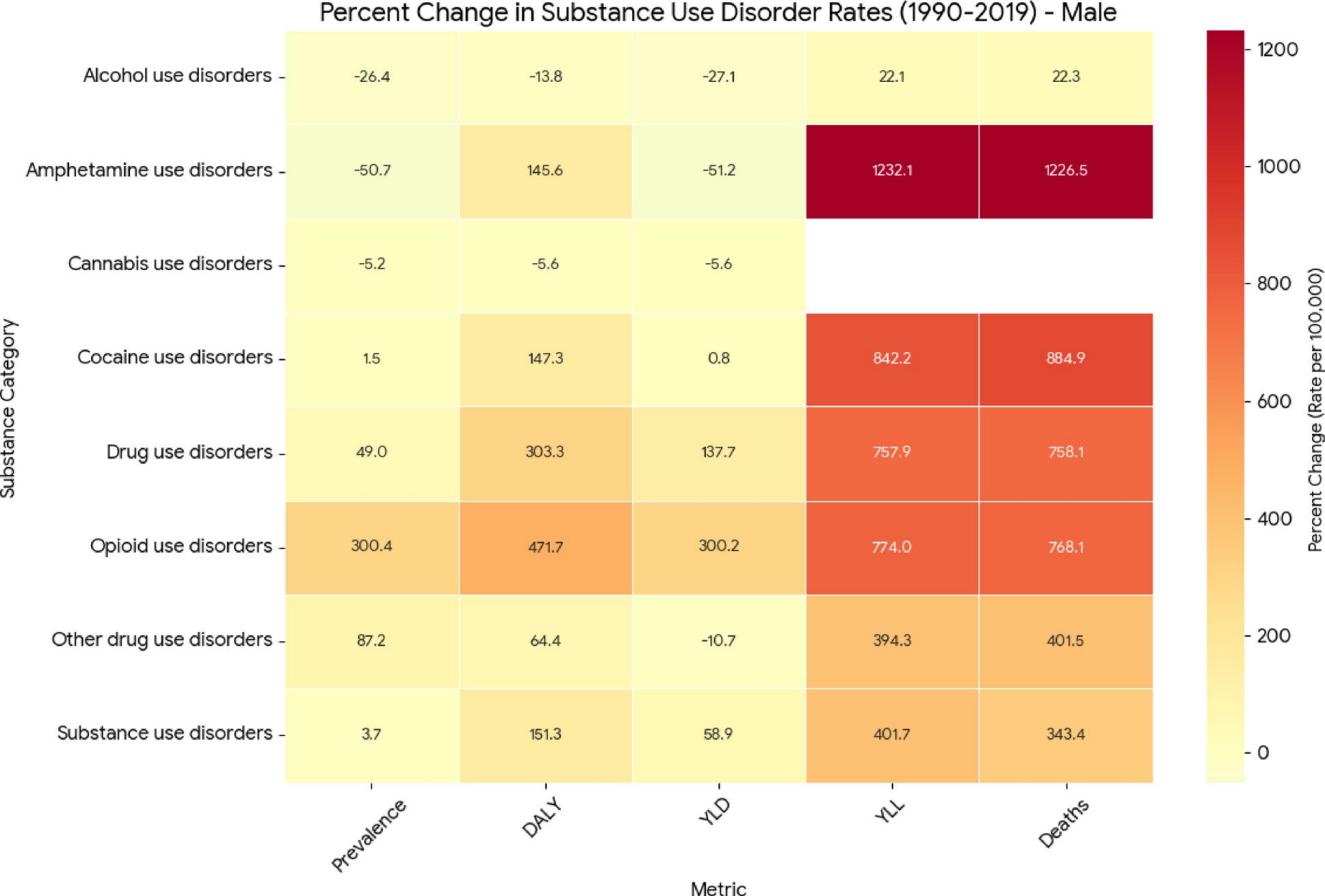
Percent change in age-standardized rates of prevalence, DALYs, YLDs, YLLs, and deaths for SUDs among males, United States, 1990–2019

To further characterize temporal changes in SUD burden, we examined percent changes in age-standardized rates of prevalence, DALYs, YLDs, YLLs, and deaths from 1990 to 2019, stratified by sex (Figs. 2 and 3). Among both females and males, relative increases were consistently greater for mortality-related outcomes, particularly YLLs and deaths, than for prevalence or YLDs across several substance categories. This pattern was most pronounced for opioid, cocaine, and amphetamine use disorders, suggesting that the burden of these disorders increased not only in frequency but also in severity over time (Supplementary Data).

### Substance use disorders related DALYs by sex and age

**Fig. 4:**
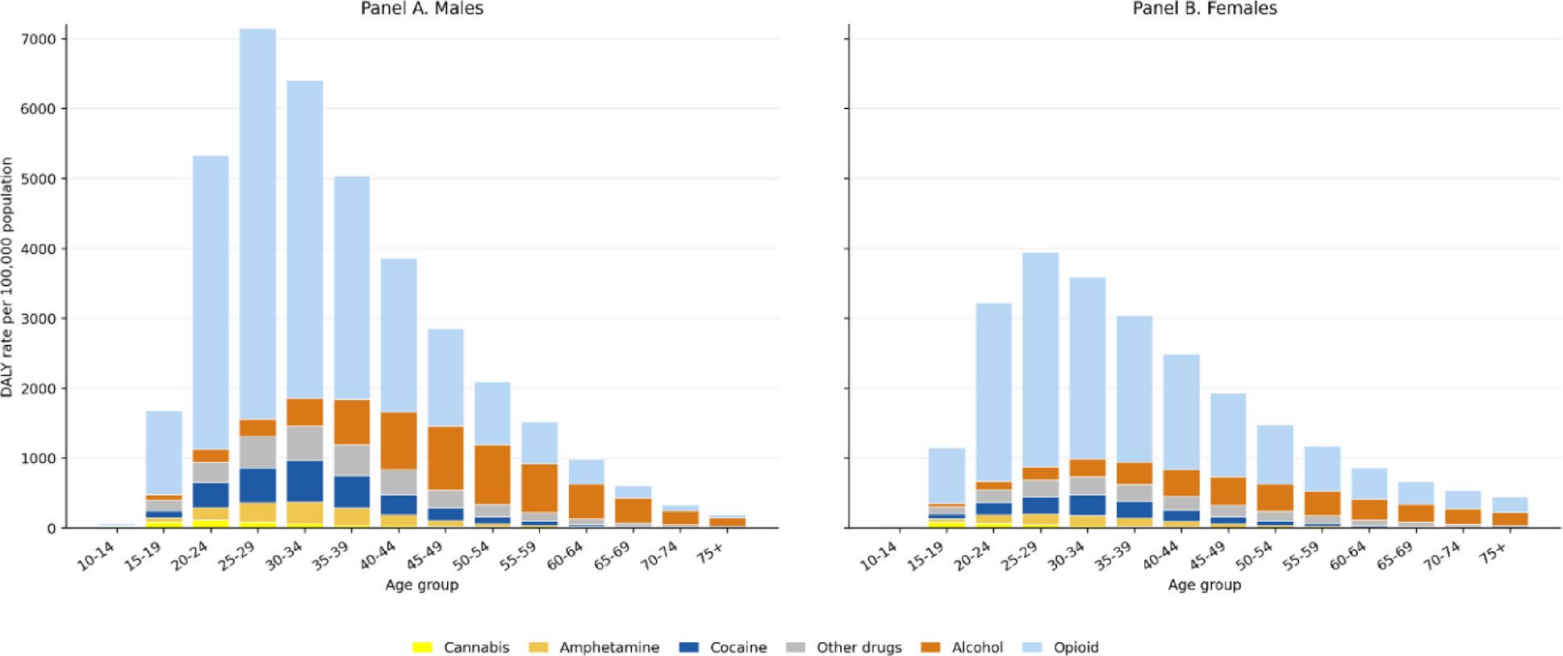
Age-specific DALY rates attributable to substance use disorders by substance category and sex in the United States, 2019. Bars represent age-specific DALY rates per 100,000 population. Total bar height reflects the overall SUD-related DALY rate within each age group

As illustrated in Fig. 4, in 2019, the DALYs attributable to SUDs peaked in early and middle adulthood, with the highest burden observed in the 20–39-year age range, among both males and females. Across all age groups, males consistently experienced higher SUD-related DALY rates than females. Opioid use disorders contributed the largest share of DALY rates in both sexes through age 64 years. The highest opioid-related burden was observed at ages 25–29 years among males (5,586.4 per 100,000) and females (3,071.6 per 100,000). Although opioid-related DALYs declined with increasing age, burdens remained comparatively elevated among females across the lifespan.

Alcohol use disorders represented the second-largest contributor overall and showed a later age distribution, peaking at ages 45–49 years among males (915.0 per 100,000) and females (402.8 per 100,000). Among males, alcohol use disorder DALY rates increased with age and exceeded opioid-related DALY rates after age 64 years, whereas among females, opioid-related DALY rates remained higher across the lifespan.

Substance-specific analysis of cocaine, amphetamine, and cannabis use disorder revealed lower overall DALY burdens but distinct age- and sex specific-patterns, with peak burden concentrated in young adulthood. Whereas cannabis use disorders accounted for the lowest DALY rates and were concentrated in adolescence and early adulthood (Fig. 4).

### Regional disparities in the burden of substance use and alcohol use disorders across the United States

**Fig. 5:**
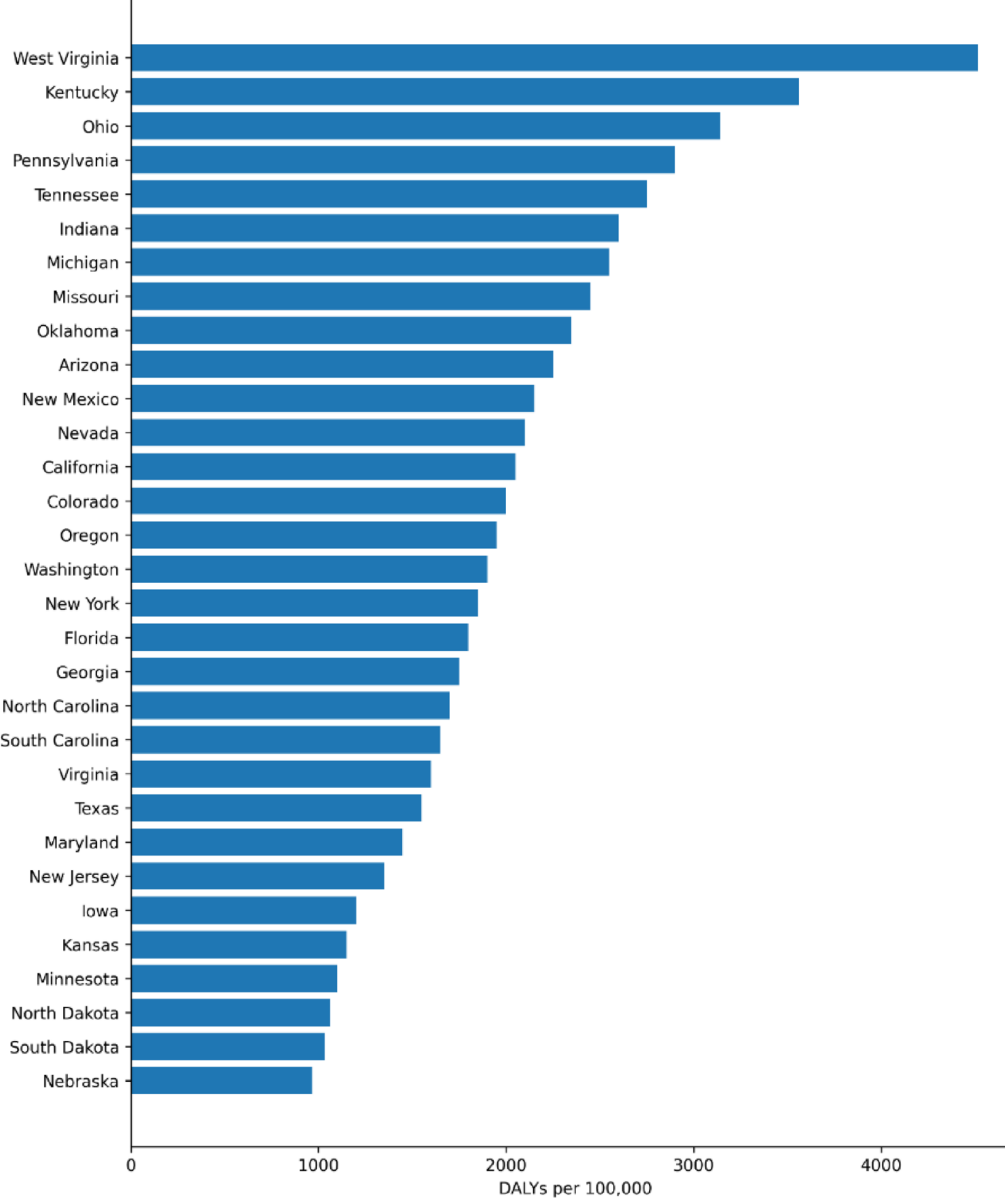
Age-standardized DALY rates attributable to drug use disorders (excluding alcohol) by state, United States, 2019

**Fig. 6:**
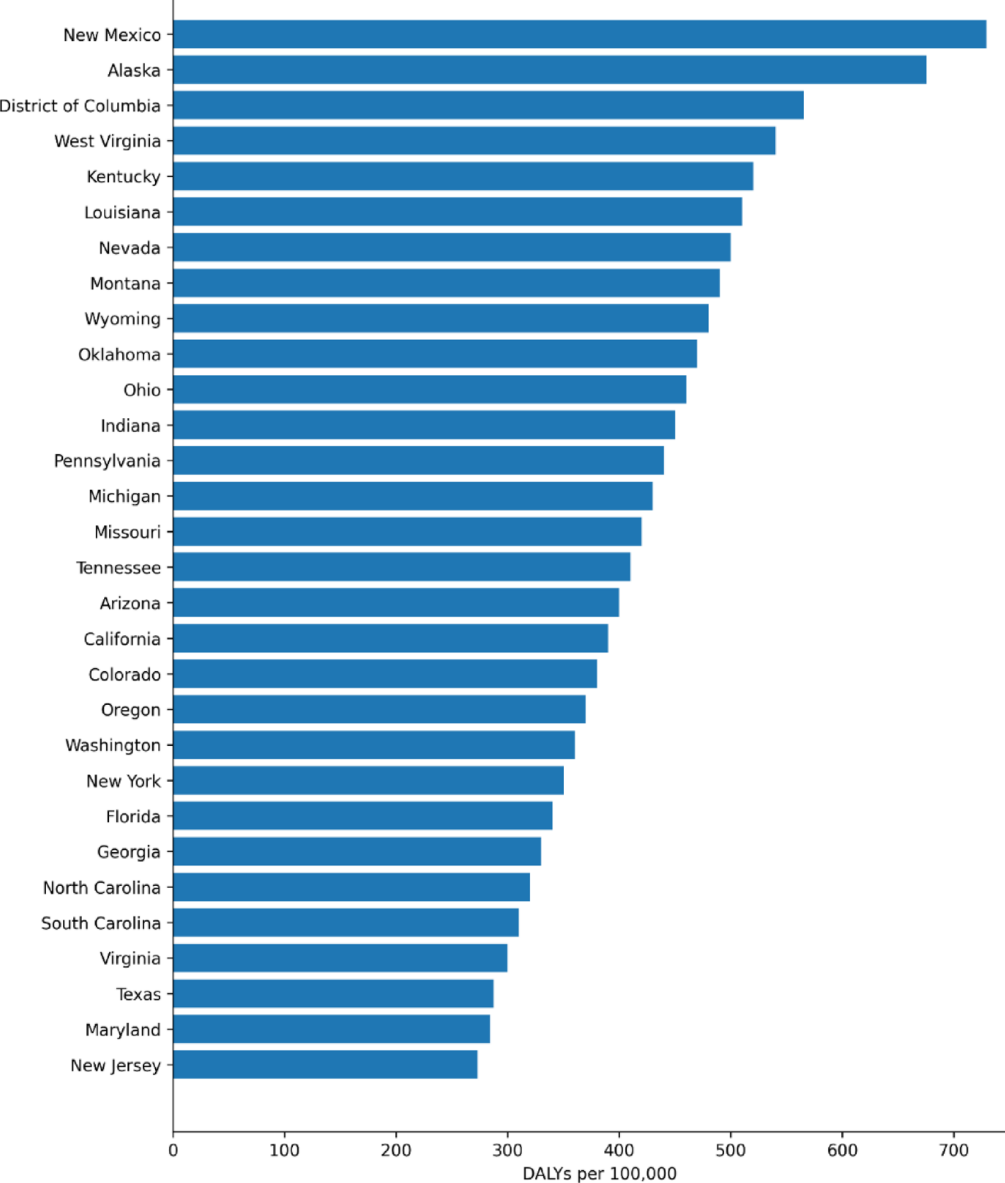
Age-standardized DALY rates attributable to alcohol use disorders by state, United States, 2019

In 2019, West Virginia had the highest age-standardized DALY rate for drug use disorders excluding alcohol (4,515.2 per 100,000 population), followed by Kentucky (3,560.7) and Ohio (3,140.4). The lowest rates were observed in Nebraska (966.3), South Dakota (1,030.6), and North Dakota (1,062.3) (Table 3; Fig. 5). Age-standardized DALY rates for alcohol use disorders were highest in New Mexico (729.1 per 100,000 population), followed by Alaska (675.5) and the District of Columbia (565.5), and were lowest in New Jersey (273.0), Maryland (284.5), and Texas (287.5) (Table 4; Fig. 6).

**Table 3:** States with the highest and lowest age-standardized DALY rates (per 100,000) for drug use disorders (excluding alcohol), both sexes, United States, 2019

States	Drug use disorder status	DALY rates	Upper 95% UI*	Lower 95% UI
West Virginia	High	4515.2	5392.5	3747.8
Kentucky	High	3560.7	4298.1	2883.6
Ohio	High	3140.4	3739.1	2612.6
North Dakota	Low	1062.3	1292.1	862.7
South Dakota	Low	1030.6	1258.6	815.1
Nebraska	Low	966.3	1198.8	761.5

*UI, uncertainty interval

**Table 4:** States with the highest and lowest age-standardized DALY rates (per 100,000) for alcohol use disorders, both sexes, United States, 2019

States	Alcohol use disorder status	DALY rates	Upper 95% UI	Lower 95% UI
New Mexico	High	729.1	877.2	596.9
Alaska	High	675.5	813.9	551.9
District of Columbia	High	565.4	701.1	445.6
Texas	Low	287.5	367.0	218.3
Maryland	Low	284.5	362.8	220.8
New Jersey	Low	272.9	345.3	210.2

### Aligning drug policies and dalys outcomes across the United States

The analysis of state-level drug and alcohol policies reveals distinct patterns between states with the lowest and highest SUD-related DALYs. Nebraska, South Dakota, and North Dakota, states with the lowest burden, have implemented multiple prevention-oriented policies, including Good Samaritan laws, PDMPs with data uploads within 24 h, interstate data sharing, and naloxone access laws [39, 41–44]. In contrast, among states with the highest SUD-related DALYs, West Virginia, Kentucky, and Ohio, policy adoption is more limited and variable, with only Kentucky supporting syringe exchange programs. To facilitate comparison of state-level policy environments, detailed summaries of drug, alcohol, and prevention-related policies and budget indicators are provided in Table 5 and Appendix Tables 1, 2, 3, 4 and 5.

**Table 5:** Summary of state-level public health policies and behavioral health budget changes for substance use disorders, selected U.S. States, 2019–2020

State	SUD burden level	Key policies enacted	Budget change (2019–2020)	Policy orientation	Notes
Nebraska	Low	PDMPs (real-time), Good Samaritan, Naloxone access, Dram Shop laws	+ 128% (from $0.97 M to $2.2 M)	Preventive	Strong multi-pronged strategy
South Dakota	Low	PDMPs, Good Samaritan, Social Host Liability	Not publicly available	Preventive	Budget info limited
North Dakota	Low	Naloxone distribution, PDMPs	Stable	Preventive	Similar to SD
Kentucky	High	Syringe access, Naloxone, partial PDMPs	–$30 M	Reactive	Fragmented enforcement
West Virginia	High	Good Samaritan, limited PDMPs use	Modest ↑	Reactive	High opioid mortality
Ohio	High	Expanded Medicaid, some naloxone distribution	Fluctuating	Mixed	Ongoing initiatives

Behavioral health budget change reflects publicly available state budget allocations for behavioral or mental health services during fiscal years 2019–2020, where available. Policy orientation is descriptive and does not imply causal effect

### Aligning alcohol policies and dalys outcomes across the United States

Examining alcohol use disorders, New Jersey emerges as a state with one of the lowest alcohol-related DALY burdens. This may reflect a broader regulatory approach that includes policies such as Dram Shop Liability laws, holding establishments accountable for serving alcohol to visibly intoxicated individuals or minors and Social Host Liability statutes that deter underage drinking in private settings [45, 46]. Similar policy environments are observed in Maryland and Texas, where restrictions such as Sunday alcohol sales bans and ignition interlock mandates for DUI offenses are in place [39]. These policies are not designed to directly treat or reduce the clinical prevalence of alcohol use disorders, but they may contribute to lowering alcohol-related injuries, impaired driving incidents, and other acute harms, which are captured in DALY estimates. Their presence may also reflect a broader state-level commitment to public health and harm reduction.

In contrast, New Mexico, Alaska, and the District of Columbia reported some of the highest alcohol-related DALY burdens. Although these jurisdictions have adopted select alcohol-related regulations, they lack several of the more comprehensive measures found in states with lower burdens. (Table 5 and Appendix Tables s1−5). These findings should be interpreted descriptively and not as evidence of causality.

## Discussion

This study provides an updated picture of the evolving burden of SUDs in the United States over the past three decades. Using GBD 2019 estimates, we examined changes in SUD-related DALYs by substance, sex, age, and state, and descriptively compared selected state policy environments and behavioral health budgets in states with the highest and lowest burden. Overall, the findings show a marked increase in SUD-related health loss between 1990 and 2019, with substantial heterogeneity across substances, age groups, sexes, and states.

### Changing trend of health burden of substance use disorders in the United States

Between 1990 and 2019, the United States experienced a 54.4% increase in prevalent SUD cases and a 213.5% increase in the age-standardized DALY rate. The largest increases were observed for opioid use disorders, followed by other drug use disorders. Cocaine and amphetamine use disorders, both categorized as stimulant use disorders, showed more modest increases in prevalence but substantially larger increases in DALY rates, indicating that the burden associated with these disorders increased disproportionately relative to case counts. In contrast, alcohol use disorder showed comparatively modest changes over time, whereas cannabis use disorder prevalence increased modestly while the age-standardized DALY rate remained stable.

These findings underscore the heterogeneous evolution of SUD burden in the United States. In particular, the marked rise in opioid-related DALYs is consistent with the changing opioid landscape, including the expansion of synthetic opioids, shifts in drug supply, and delayed or uneven policy responses [21]. The increase in DALY burden associated with stimulant use disorders also suggests that changes in severity and fatal outcomes, rather than prevalence alone, contributed meaningfully to the growing public health impact of SUDs. More broadly, the observed increase in burden likely reflects the combined influence of changing substance availability, social and structural determinants, and evolving patterns of risk exposure [1, 5, 47, 48]. 

Our findings are also consistent with national surveillance data showing rising illicit drug use and persistent substance-related harms in the United States [49, 50]. Importantly, the relative increases observed in YLLs and deaths were greater than those for prevalence or YLDs across several substance categories, particularly opioid, cocaine, and amphetamine use disorders. This pattern suggests that the growth in SUD burden over time was driven not only by increasing case counts, but also by worsening fatal outcomes.

### Sex differences in health burden of substance use disorders in the United States

Our findings indicate important sex-specific differences in the burden of SUDs. Although total age-standardized DALY rates remained higher among males than females in 2019, the increase over time was steeper among females, suggesting a narrowing but persistent disparity. In both sexes, opioid use disorders accounted for the largest increases in DALY rates, while alcohol use disorder DALY rates remained higher among males than females, with change over time was modest and differing by sex. These patterns are broadly consistent with prior research showing a substantial burden of opioid- and alcohol-related disorders in both sexes, while also underscoring important differences in level, trajectory, and substance-specific burden [47, 49–55]. 

The female increase in SUD burden warrants particular attention. Previous studies suggest that although males and females may share comparable vulnerability to developing SUDs, females may experience more rapid progression, greater relapse vulnerability, and distinct treatment [53, 56, 57] In the context of alcohol use disorders, biological differences in alcohol metabolism, lower total body water, and the telescoping phenomenon may contribute to adverse outcomes at lower levels of exposure [47]. In addition, women may face structural barriers to treatment engagement, including caregiving responsibilities, stigma, and fear of social consequences [47, 50, 58]. Together, these findings support the need for sex-responsive prevention, harm reduction, and treatment strategies.

### Health burden of substance use disorders across different age groups

Age-specific patterns in SUD burden were also pronounced. In 2019, total SUD-related DALY rates peaked in early and middle adulthood, with the highest burden observed among individuals aged 20–39 years in both sexes. Opioid use disorders accounted for the largest DALY rates in both sexes through age 64 years, with the greatest opioid-related burden observed at ages 25–29 years. Among males, alcohol use disorder DALY rates increased with age and exceeded opioid-related DALY rates after age 64 years, whereas among females, opioid-related DALY rates remained comparatively elevated across the lifespan.

Alcohol use disorders represented the second-largest contributor overall but showed a distinct and later age distribution, peaking at ages 45–49 years in both sexes. Cocaine and amphetamine use disorders contributed smaller DALY rates, with peak burden concentrated in young adulthood, whereas cannabis use disorders accounted for the lowest DALY rates and were concentrated in adolescence and early adulthood. These findings emphasize that different substances contribute to SUD burden at different stages of the life course and that prevention and treatment strategies should be calibrated accordingly.

Our age-specific findings align with previous work indicating that the consequences of substance use differ substantially across the lifespan [57]. Younger populations may be especially vulnerable because of ongoing neurodevelopment, high psychosocial stress, and co-occurring mental health risks [59–62]. At the same time, older adults may experience more substance-related medical complications, psychiatric comorbidity, and functional decline [63–67]. The later age peak observed for alcohol use disorders, particularly among males, reinforces the importance of screening and intervention strategies that extend beyond younger populations and address the distinct clinical profile of alcohol-related harm in midlife and older adulthood.

### Geographical patterns of substance use disorders across states

Our study also highlights marked state-level variation in the burden of SUDs. For drug use disorders excluding alcohol, West Virginia, Kentucky, and Ohio had the highest age-standardized DALY rates in 2019, whereas Nebraska, South Dakota, and North Dakota had the lowest. For alcohol use disorders, the highest age-standardized DALY rates were observed in New Mexico, Alaska, and the District of Columbia, while the lowest were observed in New Jersey, Maryland, and Texas. These patterns indicate that the burden of SUDs is not evenly distributed across the United States and that the geographic profile differs for alcohol-related versus other drug-related burden.

These state-level differences likely reflect a combination of contextual influences, including variation in substance availability, socioeconomic conditions, healthcare infrastructure, public health investment, and policy environments. Although this study was not designed to test the causal impact of specific policies, the geographic heterogeneity observed here supports the importance of state-level context in understanding and addressing SUD-related burden.

### Comparative policy and budget contexts across high- and low-burden states

To better contextualize state-level variation in SUD burden, we descriptively examined selected states with the highest and lowest age-standardized DALY rates attributable to drug use disorders excluding alcohol and to alcohol use disorders. Although causal inferences cannot be drawn, these comparisons provide insight into how policy environments and behavioral health funding may align with lower or higher SUD-related burden.

States with lower drug-related burden, such as Nebraska, South Dakota, and North Dakota, generally reflected more prevention-oriented policy environments, including combinations of PDMPs, naloxone access laws, Good Samaritan protections, and alcohol-related liability regulations. Nebraska also showed a notable increase in behavioral health funding during the period reviewed. Although budget data were more limited for some states, the available evidence suggests that lower-burden states may have had more stable or coordinated prevention and behavioral health infrastructure.

By contrast, states with higher drug-related burden, including West Virginia, Kentucky, and Ohio, appeared to have more variable policy and funding contexts. Although these states implemented several harm-reduction and monitoring measures, fluctuations in behavioral health investment and differences in implementation scope may help contextualize their high DALY rates. Importantly, these findings should be interpreted descriptively rather than causally.

Among states with lower alcohol-related burden, New Jersey and Maryland combined alcohol-related liability laws with regulated sales environments, whereas states with higher alcohol-related DALY rates, such as New Mexico and Alaska, experienced greater burden despite broader policy efforts. These observations suggest that the presence of policy alone may be insufficient; implementation, enforcement, treatment access, and broader structural conditions likely also contribute.

At the same time, policies intended to reduce substance-related harm may have unintended consequences. For example, prescribing restrictions or surveillance-oriented approaches may reduce some harms while also shifting risk when treatment access is limited. Similarly, punitive approaches to substance use during pregnancy may discourage engagement with prenatal care because of stigma or fear of legal repercussions. Together, these considerations underscore the importance of balancing regulatory strategies with accessible, evidence-based, and non-punitive prevention and treatment systems.

### Implications for treatment needs and access

The observed patterns in SUD burden have important implications for treatment need and service delivery. The disproportionately large contribution of opioid use disorders to total DALY burden, particularly in younger and middle-aged adults, suggests sustained demand for medications for opioid use disorder and related harm-reduction services. At the same time, the marked increase in stimulant-related DALY burden highlights ongoing unmet need for effective treatment approaches for cocaine and amphetamine use disorders, for which pharmacologic options remain limited.

Alcohol use disorders also contributed substantial burden, particularly at older ages, suggesting the need for stronger screening and treatment integration in primary care and other clinical settings serving middle-aged and older adults. More broadly, the variation observed across sex, age, substance category, and state suggests that treatment needs are unlikely to be uniform across populations. Individuals who are unhoused, incarcerated, uninsured, or living in states with constrained behavioral health infrastructure may face particularly severe barriers to timely and appropriate care.

Although treatment access and utilization were not directly assessed in this study, the alignment between population burden and state-level policy and funding context suggests that treatment capacity may be an important component of the observed disparities. These findings reinforce the importance of aligning prevention, treatment, and harm-reduction resources with the epidemiologic distribution of SUD burden.

### Is the U.S. substance use disorders profile meeting the Healthy People 2030 objectives?

Viewed against Healthy People 2030 objectives, the United States continues to experience a substantial burden of substance-related health loss, suggesting that important national targets remain unmet. This is particularly evident in the persistence of high age-standardized DALY rates, large state-level disparities, and the substantial contribution of fatal burden, especially for opioid and stimulant use disorders.

States with lower burden may offer useful contextual examples of policy and funding environments associated with more favorable outcomes; however, the present analysis does not assess whether these approaches are directly responsible for lower DALY rates. Conversely, states with persistently high burden continue to face major challenges in reducing overdose mortality and alcohol-related health loss [28, 68]. Overall, these findings suggest that progress toward Healthy People 2030 substance-related objectives will likely require coordinated, evidence-based, and adequately resourced strategies that are responsive to local epidemiology and structural conditions.

### Limitations

This study provides a descriptive epidemiological overview of trends in substance use disorders (SUDs) and state-level variation in burden; however, several limitations should be acknowledged. First, the descriptive design precludes inferential statistical testing and limits the ability to assess associations or draw causal inferences regarding SUD burden, policy environments, and behavioral health budgets. Although this approach identifies important patterns and contextual differences, future research using multivariable, longitudinal, or quasi-experimental designs could provide stronger evidence regarding these relationships.

Second, the GBD 2019 dataset did not provide sufficient disaggregation by key social determinants, including race and ethnicity, thereby limiting the ability to examine disparities across important population subgroups. This is a particularly important limitation when interpreting SUD burden through a health equity lens. Future studies incorporating more granular demographic data may provide deeper insight into differential impacts and help inform more targeted interventions.

Third, GBD estimates are model-based and derived from the synthesis of multiple data sources, including ICD-coded mortality registries, population-based surveys, and administrative datasets, each of which may be subject to measurement error, underreporting, and incomplete case ascertainment. Many of these sources rely on self-report or diagnostic coding and may underrepresent individuals who are unhoused, incarcerated, institutionalized, or not engaged with the healthcare system, likely leading to underestimation of the true burden of SUDs, particularly among marginalized populations.

Fourth, although this analysis spans a 30-year period (1990–2019), it does not examine short-term fluctuations in burden that may be associated with emergent crises, such as the rise of synthetic opioids, or with the implementation of specific state policies. More segmented time-series analyses may better capture these temporal dynamics.

Fifth, although this study incorporates state-level policy environments and behavioral health budget allocations, these comparisons are ecological and descriptive in nature. The analysis does not account for differences in policy implementation, enforcement, timing, or population reach, nor does it examine specific budget line items or actual expenditures. In addition, policy and budget environments may not align precisely with the timing of observed DALY burden, and residents living near state borders may also be influenced by neighboring state policies. A more detailed policy evaluation framework could provide greater insight into how specific regulatory mechanisms, funding priorities, and implementation strategies shape SUD-related outcomes.

Collectively, these limitations underscore the need for future research that integrates epidemiological modeling with policy analysis and equity-focused approaches to more fully inform substance use prevention and treatment strategies.

## Conclusions

Over the past three decades, the burden of SUDs in the United States has increased substantially, with marked variation across substances, age groups, sexes, and states. Opioid use disorders accounted for the largest share of DALYs, while stimulant- and alcohol-related disorders exhibited distinct age- and sex-specific patterns, highlighting the complex and evolving nature of SUD-related health loss.

Descriptive state-level comparisons suggest that more comprehensive, prevention-oriented policy environments and sustained behavioral health investment may align with lower SUD-related burden, whereas higher-burden states often reflected more variable policy and funding contexts. At the same time, the possibility of unintended consequences from regulatory and enforcement-based approaches underscores the need to balance control strategies with accessible, non-punitive, and evidence-based prevention, treatment, and harm-reduction services.

Although this study does not assess causality or treatment utilization directly, integrating epidemiologic trends with policy and funding context offers important insight into persistent disparities in SUD-related outcomes. Progress toward Healthy People 2030 substance-related objectives will likely require coordinated, equitable, and adequately resourced public health strategies tailored to differences across substances, populations, and states.

## Supplementary Information

Supplementary Material 1

Supplementary Material 2

## Data Availability

The dataset analyzed (i.e., Global Burden of Disease Study) during the current study is publicly available and downloadable from https://vizhub.healthdata.org/gbd-compare/, and also available from the corresponding author on reasonable request.

## Abbreviations

SUDs: Substance use disorders
GBD: Global burden of disease study
DALYs: Disability-adjusted life years
PDMPs: Prescription drug monitoring programs
YLDs: Years lived with disability
IHME: Institute for health metrics and evaluation
YLLs: Years of life lost
CODEm: Cause of death ensemble model
DSM-IV-TR: Diagnostic and statistical manual of mental disorders
ICD-10: International classification of diseases
TFAH: Trust for America’s health
