# Supplementary material for: The changing landscape of substance use disorders over 30 years: insights on US state disparities and policy from the global burden of disease study
Source: Popul Health Metr. 2026 Apr 20;24:38. doi: 10.1186/s12963-026-00476-3 (PMC13224409; doi:10.1186/s12963-026-00476-3)
Supplement: Supplementary file 1 — Supplementary Material 1 [file 12963_2026_476_MOESM1_ESM.docx]

**Supplementary Data:** Number of DALYs, Deaths, YLLs, YLDs, and Prevalence for Substance Use Disorders by Sex, United States, 1990 and 2019, with Percent Change (1990–2019)

| Cause | Measure | Sex | 1990 | 2019 |  |
| --- | --- | --- | --- | --- | --- |
| Alcohol use disorders | DALYs (Disability-Adjusted Life Years) | Female | 311391.9 | 472122.3 | 51.6 |
| Alcohol use disorders | DALYs (Disability-Adjusted Life Years) | Male | 730153 | 999416.8 | 36.9 |
| Alcohol use disorders | Deaths | Female | 1587.58 | 4827.06 | 204.1 |
| Alcohol use disorders | Deaths | Male | 5799.72 | 13793.32 | 137.8 |
| Alcohol use disorders | Prevalence | Female | 2588958 | 3035832 | 17.3 |
| Alcohol use disorders | Prevalence | Male | 5176603 | 5161609 | -0.3 |
| Alcohol use disorders | YLDs (Years Lived with Disability) | Female | 250666.6 | 291058.4 | 16.1 |
| Alcohol use disorders | YLDs (Years Lived with Disability) | Male | 510451 | 502076.7 | -1.6 |
| Alcohol use disorders | YLLs (Years of Life Lost) | Female | 60725.34 | 181063.9 | 198.2 |
| Alcohol use disorders | YLLs (Years of Life Lost) | Male | 219702 | 497340.1 | 126.4 |
| Amphetamine use disorders | DALYs (Disability-Adjusted Life Years) | Female | 38643.4 | 107140.2 | 177.3 |
| Amphetamine use disorders | DALYs (Disability-Adjusted Life Years) | Male | 50962.45 | 222698.2 | 337 |
| Amphetamine use disorders | Deaths | Female | 52.37 | 1179.92 | 2153 |
| Amphetamine use disorders | Deaths | Male | 137.47 | 3617.01 | 2531.1 |
| Amphetamine use disorders | Prevalence | Female | 282055.6 | 403122.7 | 42.9 |
| Amphetamine use disorders | Prevalence | Male | 336260.7 | 464391.8 | 38.1 |
| Amphetamine use disorders | YLDs (Years Lived with Disability) | Female | 35906.07 | 51549.51 | 43.6 |
| Amphetamine use disorders | YLDs (Years Lived with Disability) | Male | 43686.61 | 60817.81 | 39.2 |
| Amphetamine use disorders | YLLs (Years of Life Lost) | Female | 2737.33 | 55590.67 | 1930.8 |
| Amphetamine use disorders | YLLs (Years of Life Lost) | Male | 7275.84 | 161880.4 | 2124.9 |
| Cannabis use disorders | DALYs (Disability-Adjusted Life Years) | Female | 24146.88 | 29499.84 | 22.2 |
| Cannabis use disorders | DALYs (Disability-Adjusted Life Years) | Male | 46839.75 | 53448.4 | 14.1 |
| Cannabis use disorders | Prevalence | Female | 847203.8 | 1032923 | 21.9 |
| Cannabis use disorders | Prevalence | Male | 1622153 | 1851972 | 14.2 |
| Cannabis use disorders | YLDs (Years Lived with Disability) | Female | 24146.88 | 29499.84 | 22.2 |
| Cannabis use disorders | YLDs (Years Lived with Disability) | Male | 46839.75 | 53448.4 | 14.1 |
| Cocaine use disorders | DALYs (Disability-Adjusted Life Years) | Female | 60622.08 | 159577.1 | 163.2 |
| Cocaine use disorders | DALYs (Disability-Adjusted Life Years) | Male | 124048.4 | 404673 | 226.2 |
| Cocaine use disorders | Deaths | Female | 179.87 | 1851.37 | 929.3 |
| Cocaine use disorders | Deaths | Male | 515.45 | 5816.47 | 1028.4 |
| Cocaine use disorders | Prevalence | Female | 388064.1 | 562697.1 | 45 |
| Cocaine use disorders | Prevalence | Male | 718444.5 | 1102761 | 53.5 |
| Cocaine use disorders | YLDs (Years Lived with Disability) | Female | 51483.15 | 75015.43 | 45.7 |
| Cocaine use disorders | YLDs (Years Lived with Disability) | Male | 97473.59 | 149768.6 | 53.7 |
| Cocaine use disorders | YLLs (Years of Life Lost) | Female | 9138.93 | 84561.67 | 825.3 |
| Cocaine use disorders | YLLs (Years of Life Lost) | Male | 26574.78 | 254904.5 | 859.2 |
| Drug use disorders | DALYs (Disability-Adjusted Life Years) | Female | 350205.1 | 2447147 | 598.8 |
| Drug use disorders | DALYs (Disability-Adjusted Life Years) | Male | 613568.5 | 3822245 | 523 |
| Drug use disorders | Deaths | Female | 1558.64 | 21406.85 | 1273.4 |
| Drug use disorders | Deaths | Male | 4226.28 | 47098.46 | 1014.4 |
| Drug use disorders | Prevalence | Female | 1952963 | 5229792 | 167.8 |
| Drug use disorders | Prevalence | Male | 3157105 | 6495962 | 105.8 |
| Drug use disorders | YLDs (Years Lived with Disability) | Female | 277748.7 | 1456982 | 424.6 |
| Drug use disorders | YLDs (Years Lived with Disability) | Male | 393162.1 | 1580522 | 302 |
| Drug use disorders | YLLs (Years of Life Lost) | Female | 72456.41 | 990165.1 | 1266.6 |
| Drug use disorders | YLLs (Years of Life Lost) | Male | 220406.3 | 2241723 | 917.1 |
| Opioid use disorders | DALYs (Disability-Adjusted Life Years) | Female | 212304 | 2067728 | 873.9 |
| Opioid use disorders | DALYs (Disability-Adjusted Life Years) | Male | 364233.2 | 3001250 | 724 |
| Opioid use disorders | Deaths | Female | 1147.07 | 16839.01 | 1368 |
| Opioid use disorders | Deaths | Male | 3138.85 | 34957.47 | 1013.7 |
| Opioid use disorders | Prevalence | Female | 400673.9 | 3205107 | 699.9 |
| Opioid use disorders | Prevalence | Male | 485114 | 3159443 | 551.3 |
| Opioid use disorders | YLDs (Years Lived with Disability) | Female | 160448.3 | 1289167 | 703.5 |
| Opioid use disorders | YLDs (Years Lived with Disability) | Male | 200289.5 | 1307510 | 552.8 |
| Opioid use disorders | YLLs (Years of Life Lost) | Female | 51855.76 | 778560.8 | 1401.4 |
| Opioid use disorders | YLLs (Years of Life Lost) | Male | 163943.7 | 1693740 | 933.1 |
| Other drug use disorders | DALYs (Disability-Adjusted Life Years) | Female | 14488.71 | 83202.15 | 474.3 |
| Other drug use disorders | DALYs (Disability-Adjusted Life Years) | Male | 27484.75 | 140176 | 410 |
| Other drug use disorders | Deaths | Female | 179.34 | 1536.56 | 756.8 |
| Other drug use disorders | Deaths | Male | 434.51 | 2707.52 | 523.1 |
| Other drug use disorders | Prevalence | Female | 53878.51 | 109523.7 | 103.3 |
| Other drug use disorders | Prevalence | Male | 44740.95 | 82419.78 | 84.2 |
| Other drug use disorders | YLDs (Years Lived with Disability) | Female | 5764.32 | 11750.09 | 103.8 |
| Other drug use disorders | YLDs (Years Lived with Disability) | Male | 4872.72 | 8977.41 | 84.2 |
| Other drug use disorders | YLLs (Years of Life Lost) | Female | 8724.39 | 71452.06 | 719 |
| Other drug use disorders | YLLs (Years of Life Lost) | Male | 22612.03 | 131198.6 | 480.2 |
| Substance use disorders | DALYs (Disability-Adjusted Life Years) | Female | 661597 | 2919270 | 341.2 |
| Substance use disorders | DALYs (Disability-Adjusted Life Years) | Male | 1343721 | 4821662 | 258.8 |
| Substance use disorders | Deaths | Female | 3146.22 | 26233.91 | 733.8 |
| Substance use disorders | Deaths | Male | 10026 | 60891.78 | 507.3 |
| Substance use disorders | Prevalence | Female | 4478826 | 8122166 | 81.3 |
| Substance use disorders | Prevalence | Male | 8144934 | 11368737 | 39.6 |
| Substance use disorders | YLDs (Years Lived with Disability) | Female | 528415.3 | 1748041 | 230.8 |
| Substance use disorders | YLDs (Years Lived with Disability) | Male | 903613.1 | 2082599 | 130.5 |
| Substance use disorders | YLLs (Years of Life Lost) | Female | 133181.8 | 1171229 | 779.4 |
| Substance use disorders | YLLs (Years of Life Lost) | Male | 440108.4 | 2739063 | 522.4 |
