# Supplementary material for: The changing landscape of substance use disorders over 30 years: insights on US state disparities and policy from the global burden of disease study
Source: Popul Health Metr. 2026 Apr 20;24:38. doi: 10.1186/s12963-026-00476-3 (PMC13224409; doi:10.1186/s12963-026-00476-3)

**Appendix Tables 1–5**

**
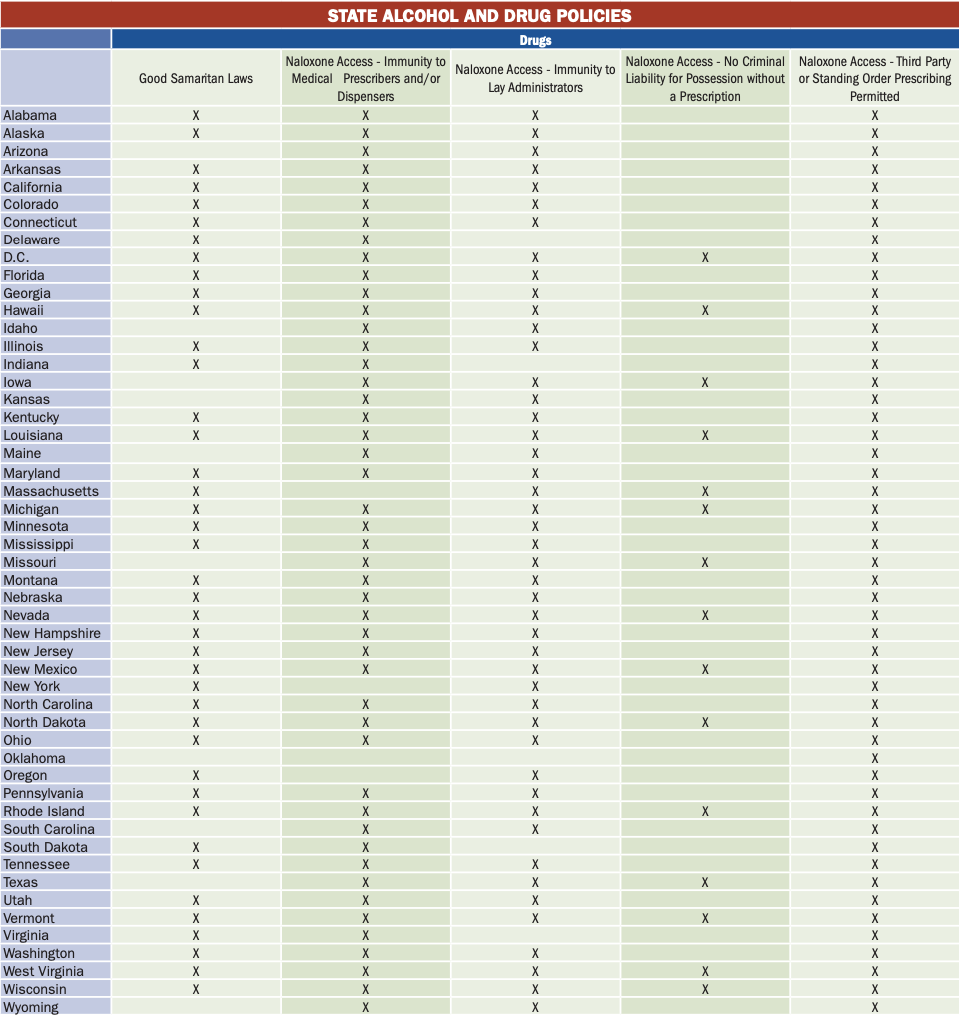
Table 1.** State Drug Policies, including Good Samaritan Laws and Naloxone Access *

*Segal, L. M., De Biasi, A., Mueller, J. L., May, K., & Warren, M. (2017). Pain in the Nation: The Drug, Alcohol, and Suicide Crises and the Need for a National Resilience Strategy Retrieved from <https://www.tfah.org/report-details/pain-in-the-nation/>

**Table 2**. State Drug Policies, including Prescription Drug Monitoring Programs *


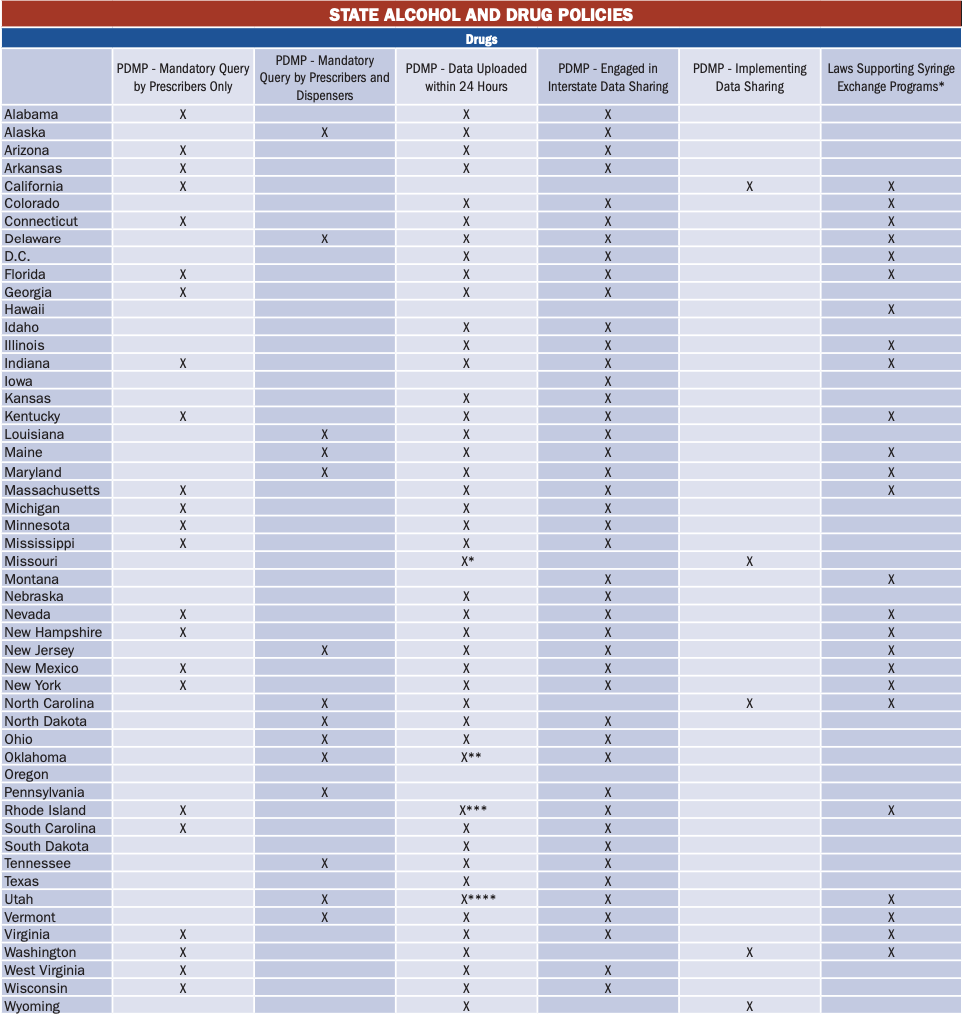


* Segal, L. M., De Biasi, A., Mueller, J. L., May, K., & Warren, M. (2017). Pain in the Nation: The Drug, Alcohol, and Suicide Crises and the Need for a National Resilience Strategy Retrieved from <https://www.tfah.org/report-details/pain-in-the-nation/>

**
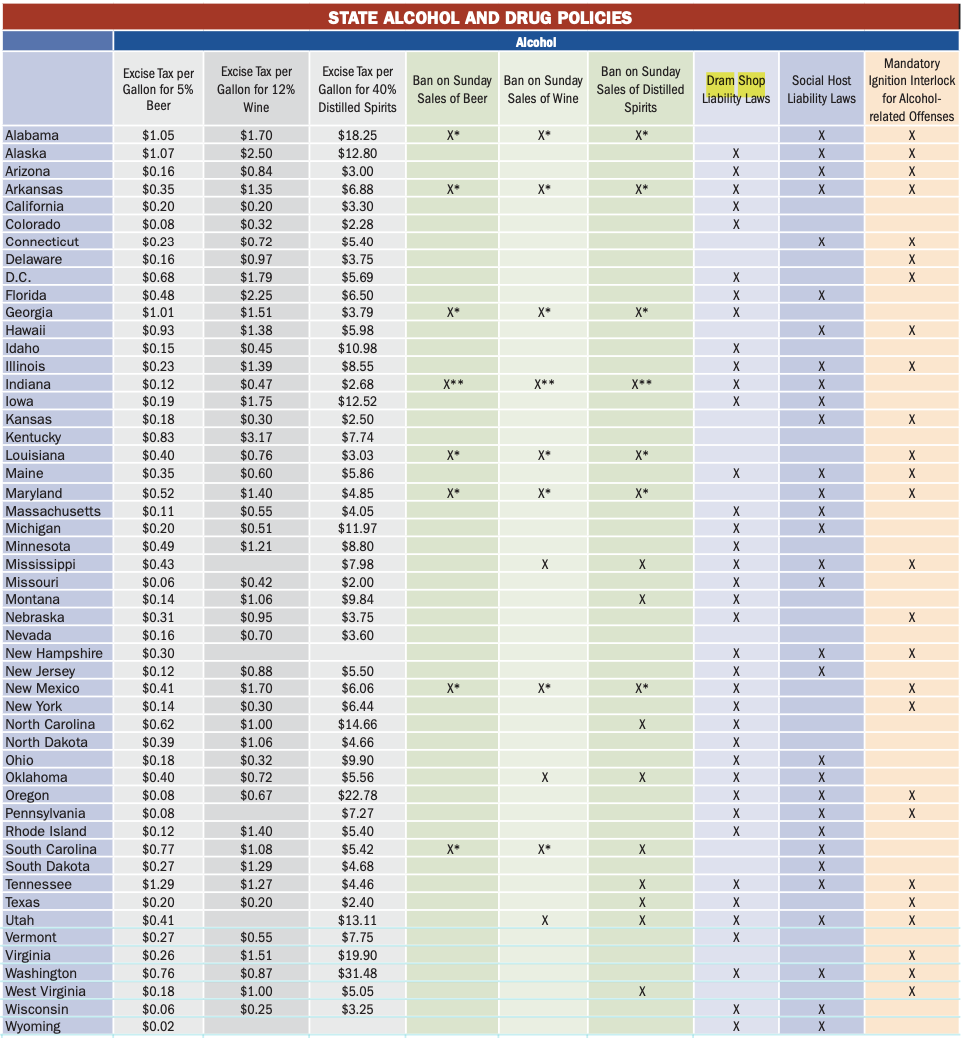
Table 3.** State Alcohol Policies *

* Segal, L. M., De Biasi, A., Mueller, J. L., May, K., & Warren, M. (2017). Pain in the Nation: The Drug, Alcohol, and Suicide Crises and the Need for a National Resilience Strategy Retrieved from <https://www.tfah.org/report-details/pain-in-the-nation/>

**Table 4.** Prevention Policy Indicators, Including State Mental Health Budget Status *


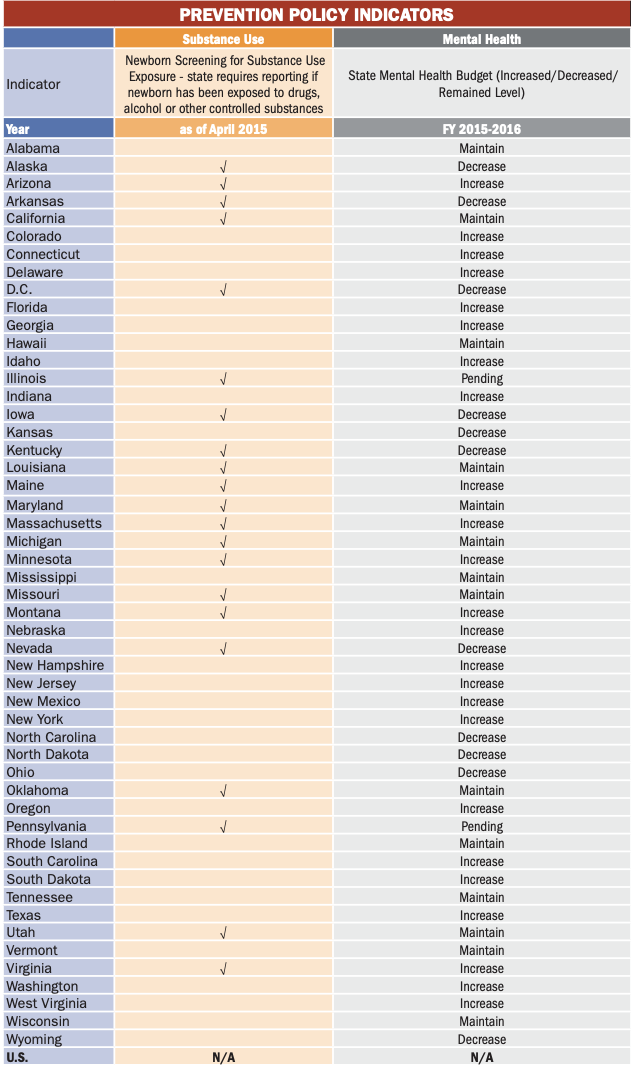


* Segal, L. M., De Biasi, A., Mueller, J. L., May, K., & Warren, M. (2017). Pain in the Nation: The Drug, Alcohol, and Suicide Crises and the Need for a National Resilience Strategy Retrieved from <https://www.tfah.org/report-details/pain-in-the-nation/>

**Table 5.** Overview and Objectives of Healthy People 2030
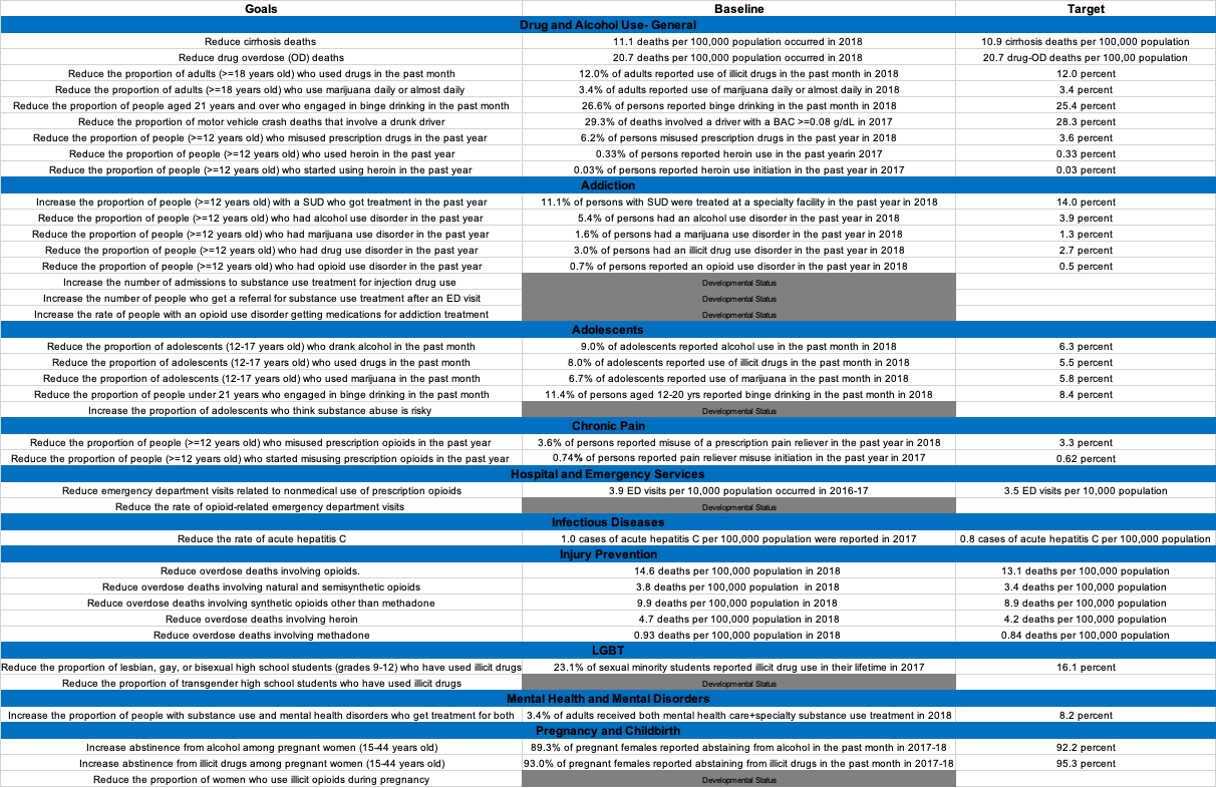

Supplement: Supplementary file 2 — Supplementary Material 2 [file 12963_2026_476_MOESM2_ESM.docx]
